# Texture discriminability in monkey inferotemporal cortex predicts human texture perception

**DOI:** 10.1152/jn.00532.2014

**Published:** 2014-09-10

**Authors:** Kalathupiriyan A. Zhivago, Sripati P. Arun

**Affiliations:** Centre for Neuroscience, Indian Institute of Science, Bangalore, India

**Keywords:** object recognition, perception, texture, inferior temporal cortex, neurophysiology

## Abstract

Shape and texture are both important properties of visual objects, but texture is relatively less understood. Here, we characterized neuronal responses to discrete textures in monkey inferotemporal (IT) cortex and asked whether they can explain classic findings in human texture perception. We focused on three classic findings on texture discrimination: *1*) it can be easy or hard depending on the constituent elements; *2*) it can have asymmetries, and *3*) it is reduced for textures with randomly oriented elements. We recorded neuronal activity from monkey inferotemporal (IT) cortex and measured texture perception in humans for a variety of textures. Our main findings are as follows: *1*) IT neurons show congruent selectivity for textures across array size; *2*) textures that were easy for humans to discriminate also elicited distinct patterns of neuronal activity in monkey IT; *3*) texture pairs with asymmetries in humans also exhibited asymmetric variation in firing rate across monkey IT; and *4*) neuronal responses to randomly oriented textures were explained by an average of responses to homogeneous textures, which rendered them less discriminable. The reduction in discriminability of monkey IT neurons predicted the reduced discriminability in humans during texture discrimination. Taken together, our results suggest that texture perception in humans is likely based on neuronal representations similar to those in monkey IT.

texture is an important property of objects, but it has received relatively little attention compared with shape. Classic studies of texture perception have attempted to explain the ease with which humans identify a texture border as a function of the constituent texture elements. These studies have reported three important phenomena in texture perception (Fig. 1). First, some textures are easy to discriminate whereas others are hard (Fig. 1*A*) (Beck 1972; Julesz 1986). Second, there are asymmetries in texture perception: finding a texture A embedded in B can sometimes be easier than finding B in A (Fig. 1*B*) (Gurnsey and Browse 1987; Williams and Julesz 1992). Third, when the elements of two textures are oriented randomly, finding their border becomes hard in some cases but not others (Fig. 1*C*) (Beck 1972; Julesz 1986; Malik and Perona 1990). While there have been attempts to explain texture discrimination using image properties (Beck 1972; Julesz 1981a,b; Bergen and Julesz 1983; Julesz 1984) and computational models (Malik and Perona 1990), there is no comprehensive account of these phenomena and their underlying neuronal correlates.

**Fig. 1.**
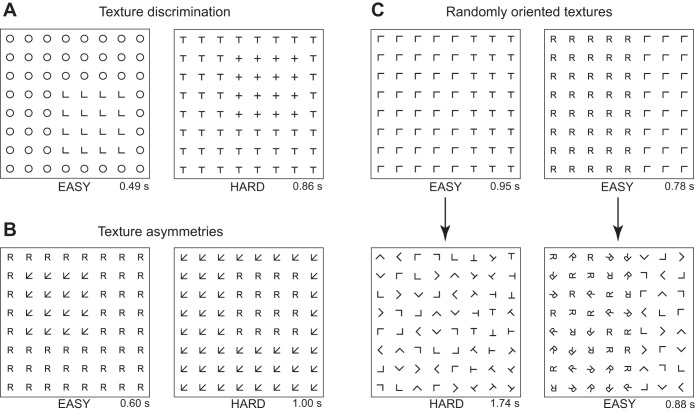
Three classic findings in human texture perception. *A*: texture discrimination: some textures are easier to distinguish than others. For instance, it is easier to find a texture of Ls within a texture made of Os (*left*) than to find a patch of +s within a texture made of Ts. Numbers at the *bottom right* are average times taken by humans to locate the target texture. *B*: texture asymmetry: it is sometimes easier to find a texture A inside a texture B than vice versa. For instance, it is easier to locate arrows among Rs (*left*) than to locate Rs among arrows (*right*). *C*: randomly oriented textures: some textures become harder to distinguish when their elements are randomly oriented. For instance, the border between the L and T textures becomes extremely hard to find when the Ls and Ts are randomly oriented (*left*), but the border between the Rs and Ls becomes only slightly harder to find (*right*).

Our goal was to investigate whether the above phenomena in human texture perception can be understood in terms of neuronal activity in monkey visual cortex. We selected the monkey inferior temporal cortex (IT) as a likely substrate because its neurons are selective for both shape and texture (Kovacs et al. 2003; Köteles et al. 2008), and at the population level, its object representations are closely linked to behavior in monkeys (Op de Beeck et al. 2001; Sigala et al. 2002; Afraz et al. 2006; Verhoef et al. 2012) and humans (Sripati and Olson 2009; Sripati and Olson 2010; Kriegeskorte et al. 2008). A second goal of our study was to characterize how IT neurons represent discrete textures. This issue remains largely unexplored: previous studies have characterized how IT neurons respond to shapes filled with a variety of textures (Kovacs et al. 2003; Köteles et al. 2008) but have not elucidated how responses to a texture are related to the local shapes in a texture. This question is difficult to answer using natural textures because they contain local features that cannot be manipulated without disrupting other features or their overall arrangement. Discrete textures, on the other hand, permit easy manipulation of local shape. This allowed us to ask the basic question: do IT neurons respond fundamentally differently to the same shape when presented in isolation vs. as a large texture field?

We performed a total of four experiments: two neurophysiological experiments on monkey IT cortex and two behavioral experiments on humans. In *experiment 1*, we characterized the responses of IT neurons to discrete textures at various array sizes. IT neurons showed congruent tuning for textures across array size. Thus texture selectivity in IT neurons, at least for discrete textures, was largely explained using selectivity for shape. In *experiment 2*, we measured texture discrimination in humans for the same textures. We found that neuronal discriminability in monkey IT accurately predicted texture discrimination performance in humans. Furthermore, asymmetries in human texture discrimination were predicted using the population variation in firing rate across IT neurons. In *experiment 3*, we recorded neuronal responses to randomly oriented and uniform textures. Neuronal responses to random textures were predicted by the average of responses to uniformly oriented textures, leading to reduced discriminability. In *experiment 4*, we found parallel reductions in texture discriminability for randomly oriented textures in humans. Taken together, our results show that texture representations in monkey IT neurons predict human texture discrimination.

## METHODS

### Neurophysiology

All experiments were performed according to a protocol approved by the Institutional Animal Ethics Committee of the Indian Institute of Science, Bangalore and the Committee for the Purpose of Control and Supervision of Experiments of Animals, Government of India. These procedures also conform to the American Physiological Society's *Guiding Principles in the Care and Use of Vertebrate Animals in Research*. Single-unit activity was recorded from the inferior temporal cortex of two naïve adult male monkeys (*Macaca radiata;* laboratory designations Ro and Xa; aged ∼12 yr). Each monkey underwent two surgeries under anesthesia: the first, to implant a titanium head post for behavioral training on fixation tasks, and the second, to implant a recording chamber (Crist Instruments) positioned vertically above their left hemisphere IT. The location of the recording chamber was determined using structural MRI to be centered over the anterior portion of left IT. The actual chamber locations (A14, L19 mm in Ro and A18, L18 mm in Xa) corresponded to A19, L19 mm in a standard rhesus monkey atlas (Saleem and Logothetis 2007). These locations were subsequently verified during recording using phasic transitions and anatomical landmarks during recording and also using postmortem histology in one monkey (Xa).

On each day of recording, a tungsten microelectrode (FHC) was lowered into the brain using a micromanipulator (Narishige) with the aid of a stainless steel guide tube. The electrode was then advanced until phasic visual responses were observed. Action potentials and field potentials were recorded using a commercial amplifier (Omniplex system; Plexon). Action potentials were initially isolated using a high-pass filter (4-pole Butterworth filter, cutoff frequency: 250 Hz) and then sorted offline into putative individual units using a spike sorting software based on waveform and cluster analysis (OfflineSorter; Plexon).

#### Behavioral task.

All aspects of the behavioral task were under control of a computer running Cortex software (NIMH DOS Cortex). Eye position was monitored using an infrared 250-Hz eye tracker system (ETL-250; ISCAN). Stimuli were presented on an LCD monitor (VX2268wm; ViewSonic; 120-Hz refresh rate). Monkeys were trained to perform a fixation task. Each trial began with the appearance of a red fixation dot (0.2° diameter). Once the monkey began to fixate on the dot, a series of eight images appeared in succession. Each stimulus lasted 200 ms with an interstimulus interval of 200 ms. The interstimulus interval contained a blank screen with just the fixation dot. On successfully maintaining fixation within a 3° window of the fixation dot throughout the trial, the monkey received a juice reward (post hoc analyses revealed that the animals' gaze stayed close to the fixation dot during correct trials, with a standard deviation of 0.24 and 0.32°, respectively, along the horizontal and vertical). Trial order was random and error trials (fixation breaks and failures to begin fixating) were repeated after a random number of trials. The average performance of the animals was 72% (Ro = 68% and Xa = 76%). To confirm that behavioral performance on the task did not influence our results, we repeated the key analyses in both *experiments 1* and *3* on recordings in which the animals had high and low performance. We obtained qualitatively similar results.

#### Experiment 1 (uniform textures).

**Fig. 2.**
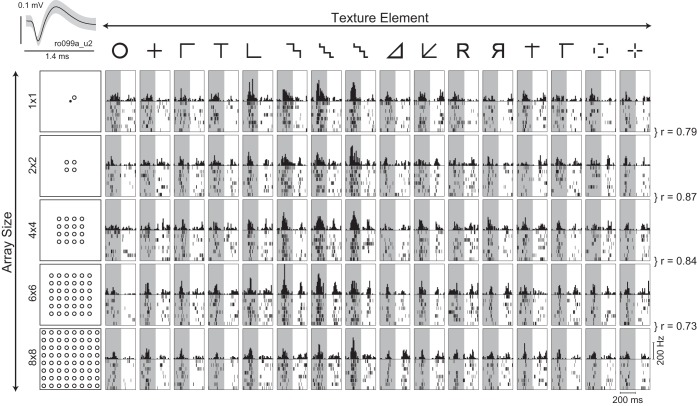
Responses of an example inferotemporal (IT) neuron to discrete textures (*experiment 1*). A total of 16 textures (*columns*) at 5 array sizes (*rows*) were shown as stimuli (the actual stimuli were white against a black background). Each grid location contains a plot with the peristimulus time histogram (in 10-ms bins) following image onset, and the rasters below indicate the spikes evoked across multiple trials. Texture elements are along the topmost row, and the arrays for a sample shape (“O”) are shown along the leftmost column. The shaded region corresponds to the image presentation period. The dot in the 1 × 1 array indicates a red fixation dot (shown only for scale, the dot was never shown with the stimuli). Correlations between responses to shapes at adjacent array sizes (e.g., 1 × 1 vs. 2 × 2, 4 × 4 vs. 6 × 6) are indicated at the extreme right (all correlations were significant *P* < 0.005). *Top left inset*: average action potential waveform for this neuron (ro099a_u2).

The goal of this experiment was to characterize how IT neurons respond to discrete textures at various array sizes. Stimuli consisted of textures made of 16 basic shapes presented as five possible texture arrays: 1 × 1, 2 × 2, 4 × 4, 6 × 6, and 8 × 8 (see Fig. 2). The shapes were based on studies of texture perception in humans (Krose 1986; Gurnsey and Browse 1987; Malik and Perona 1990). Arrays were constructed such that the locations of all elements (across arrays) fell on a fixed 8 × 8 grid (see Fig. 2). Each texture element measured 0.5 × 0.5°, and the center-to-center spacing between elements was 1°. To avoid large changes in image size within a trial, textures of each array size were presented in separate blocks in random order. The order of blocks was randomized across neurons. We recorded from a total of 168 IT neurons (*n* = 136 and 32 neurons from Ro and Xa, respectively) in this experiment.

To confirm that the key results of this experiment were the same across both animals, we repeated the analysis using the data from each monkey separately. The neuronal-behavioral correlation (see Fig. 5*A*) was statistically significant for the data from each monkey as well (*r* = 0.65, *P* < 0.00005 for Ro and *r* = 0.43, *P* < 0.00005 for Xa).

#### Experiment 3 (randomly oriented textures).

The goal of this experiment was to characterize neuronal responses to uniform textures (containing only one orientation) and to textures containing a single element at random orientations inside the texture. We chose a subset of four texture elements from *experiment 1* (denoted as T, L, M, and R; see Fig. 6). These textures were presented at the 8 × 8 array size with the elements in 8 orientations (0, 45, 90, 135, 180, 225, 270, and 315° clockwise from the vertical), which yielded 64 stimuli. In addition, we created two 8 × 8 arrays of randomly oriented textures using each texture element, such that each row or column of the 8 × 8 array contained all possible eight orientations of a given texture element. This yielded a total of 8 stimuli (4 elements × 2 random textures). Stimuli were presented in two blocks: uniform textures and random textures. Images within a trial were ordered such that no two successive textures contained the same texture element. All other parameters were identical to *experiment 1*. We recorded from a total of 120 visually responsive neurons (101 from Ro and 19 from Xa) in this experiment.

To confirm that the key results of this experiment were the same across both animals, we repeated these analysis using the data from each monkey separately. The neuronal-behavioral correlation (see Fig. 6*C*) was statistically significant for the data from each monkey as well (*r* = 0.78, *P* < 0.005 for Ro, and *r* = 0.78, *P* < 0.005 for Xa).

### Human Psychophysics

All participants gave written informed consent to an experimental protocol approved by the Institute Human Ethics Committee of the Indian Institute of Science. Subjects were seated in front of a monitor ∼50 cm away that was under control of custom programs written in Matlab using Psychtoolbox (Brainard 1997) and were instructed to respond on each trial by making a key press. Subjects were free to move their head and eyes during the task.

#### Experiment 2 (uniform textures).

The goal of this experiment was to measure texture discrimination performance in humans using stimuli identical to those in *experiment 1*. A total of 16 naïve subjects (12 male, aged 20–30 yr) with normal or corrected-to-normal vision participated in the experiment. On each trial a 16 × 16 array of items (measuring 16° square, element size 0.5° as in the neuronal experiments) was presented on the screen along with a red vertical dividing line down the middle. Of the 256 items in the 16 × 16 array, a 4 × 4 patch contained the target texture and the surrounding elements formed the distracter texture. Subjects were required to report the side of the divider line on which the target was present using a key press (“Z” for left and “M” for right) within 7.5 s of stimulus presentation (failing which the trial was aborted). The target texture always appeared within the inner 14 × 14 part of the large array, so that its items were always surrounded by the distracter texture.

Subjects discriminated all possible texture pairs over the course of the experiment: we used all 16 textures from *experiment 1*, which yielded ^16^C_2_ = 120 pairs of textures. Each texture pair (A, B) was presented with either item as target, and the target texture appeared on the left or right side. Each unique condition was repeated three times. This yielded a total of 1440 trials in the entire experiment (120 pairs × 2 A/B as target × 2 left/right × 3 repetitions). Trial order was random. Error and aborted trials were repeated after a random number of other trials.

#### Experiment 4 (randomly oriented textures).

The goal of this experiment was to measure texture discrimination performance in humans for textures with randomly oriented elements. A total of eight naïve subjects (5 male, aged 20–30 yr) with normal or corrected-to-normal vision performed the experiment. All experiment procedures were identical to *experiment 2*, and only the differences are detailed below.

The texture array on each trial consisted of an 8 × 8 array (measuring 8° square; items and spacing as before) containing two textures that had a red vertical border as before. There were five columns of one texture and three columns of the other texture that formed an 8 × 8 array with the same dimensions and spacing as in *experiment 1*. Subjects were required to report the side (left/right) on which the texture boundary was located using a key press. The textures comprised 4 basic elements that could be in 8 possible orientations, resulting in a total of 32 textures. Subjects performed texture discrimination on all possible texture pairs (^32^C_2_ = 496 pairs). Each pair (A, B) was repeated two times with either A or B as target (with boundary chosen on the left or right). In addition, they also saw all possible pairs (^4^C_2_ = 6) of random textures. Each such pair (A, B) was repeated eight times (4 times each with boundary on left or right). This resulted in a total of 1,040 correct trials (992 uniform texture trials and 48 random texture trials) for each subject.

### Data Analysis

#### Unbiased estimates of ANOVA effect strength.

To investigate how texture and array size modulated neuronal responses, we performed an ANOVA on the firing rate of each neuron (in a window 50–200 ms after image onset) with texture (16 levels) and array size (5 levels) as factors (see Fig. 4*A*). In the ANOVA, the modulation (or coefficient) corresponding to each texture is taken as the difference between the average response elicited by it and the global average response. Thus the modulation can be positive or negative depending on the texture. To assess the overall modulation due to texture, we could have taken the average positive minus the negative coefficients in the ANOVA. However, this measure will be biased because it will be always positive even for random noise. To obtain an unbiased estimate of the texture main effects, we used a split-half analysis: we separated the available trials into two halves, calculated the sign of the ANOVA texture effect coefficients using one-half of the data, and multiplied them by ANOVA texture effect coefficients in the other half of the data and took the average of these numbers. We repeated this procedure twice: once with the effect sign from the even numbered trials and effect magnitude from odd-numbered trials, and once with the sign from odd, and magnitude from even-numbered trials, and then averaged the two split-half estimates. This final estimate is now an unbiased estimate of the texture modulation because random noise will produce unrelated fluctuations in both halves of the data. In contrast, systematic modulations due to texture will result in large estimates because the magnitude of texture modulations in both halves of the data will be correlated. We performed analogous analyses to obtain unbiased estimates of the array size and interaction effects.

#### d-Prime measures for neurons and behavior.

We performed a d-prime analysis on both neuronal and behavioral data to confirm the robustness of the neuronal-behavioral correlation. For each texture pair, we calculated a measure of discriminability based on signal detection theory, using the formula d-prime = z(H) − z(FA), where z is the inverse of the cumulative standard normal distribution and H represents the hit probability, and FA represents the false alarm probability. In the case of behavioral data, we pooled the responses from all subjects and all trials for a given texture pair and denoted (without loss of generality) the left target as the signal to be detected. Thus the hit probability represented the fraction of trials in which the subject correctly judged the oddball texture to be on the left. The false alarm rate was then the fraction of right-target trials that the subject incorrectly declared as being on the left.

In the case of neuronal data, for each neuron, we took the firing rates on all trials corresponding to the two textures. For each of these trials, we used a linear classifier (trained on the remaining trials) to determine which texture was presented given the response observed on that trial. This yielded a pattern of correct and incorrect responses, which represents the performance of an ideal observer listening to the responses of this neuron. We then calculated a d-prime measure for each neuron and averaged this across all neurons to obtain the average d-prime across IT.

#### Unbiased estimates of texture asymmetry.

In *experiment 2*, for each pair of textures (A, B), subjects performed trials in which they had to distinguish texture A within a larger patch of texture B or vice versa (see Fig. 5*B*). We then measured the discriminability of A from B (d_AB_) as the reciprocal of the time taken by subjects to perform this task (Sripati and Olson 2010; Arun 2012). A direct estimate of the asymmetry would be to simply take the absolute difference |d_AB_ − d_BA_|, where d represents discriminability and AB and BA indicate the two searches with A or B as target. However, exactly as detailed above, such an estimate would be biased because even random noise would yield a positive value. To correct for this bias, we separated the subjects' data into two halves. Using one-half of the data, we identified the target that was more discriminable compared with the other. We then calculated the difference in discriminability d_AB_ − d_BA_ from the other half of the data, where A and B are now the target identified as easy and hard using the first half of the data. We repeated this procedure twice by identifying the easy and hard targets using the odd numbered subjects and calculating the discriminability using the even-numbered subjects and vice versa. By averaging the two estimates, we obtained an unbiased estimate of the magnitude of the texture discrimination asymmetry.

## RESULTS

We recorded single-unit activity from the left inferotemporal cortex of two macaque monkeys while they viewed a variety of discrete textures in a fixation task. We also measured texture discrimination performance of humans using the same textures. Because no previous study has characterized how IT neurons represent discrete textures, we first set out to systematically characterize texture representations in IT neurons (*experiment 1*). We then sought to establish whether texture perception in humans can be understood using texture representations in IT (*experiment 2*). In *experiment 3*, we characterized neuronal responses to randomly oriented textures and how they are related to uniform texture responses. In *experiment 4*, we asked whether the reduced discriminability for randomly oriented textures in humans can be explained using texture representations observed in IT neurons.

### Experiment 1: Uniform Textures (Neurophysiology)

**Fig. 3.**
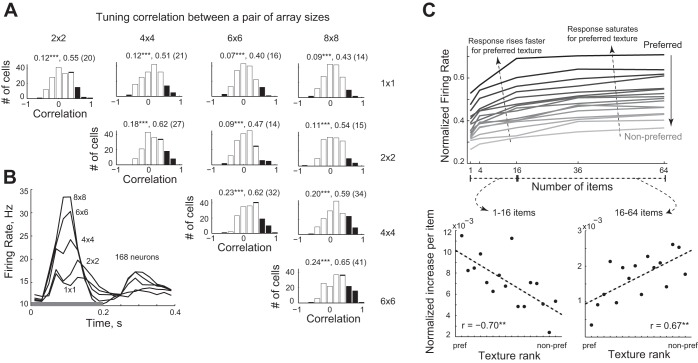
Discrete texture representation in IT neurons. To investigate how neuronal selectivity for textures changes across array sizes, we calculated the correlation between texture responses to each pair of array sizes. *A*: distribution of correlation coefficients across all visual neurons (white bars: all neurons, dark bars: significant correlations). The numbers above represent (in that order) the average correlation, average of the significant correlations and the number of neurons that had a significant correlation. Here and throughout: *P < 0.05, **P < 0.005, statistical significance. B: time course of the population response (averaged across all textures for all 168 neurons) for each array size as a function of time (in bins of 10 ms). The shaded bar above the *x*-axis represents the image presentation period. *C*, *top*: average normalized responses (across 168 neurons) plotted as a function of array size for textures ranked from preferred (rank 1) to nonpreferred (rank 16) texture. *C*, *bottom left*: The per item increase in neuronal response for fewer items (for arrays 1 × 1, 2 × 2 and 4 × 4) decreases from preferred to nonpreferred, yielding a significant negative correlation. *C*, *bottom right*: The per item increase in response for larger arrays (arrays 4 × 4, 6 × 6 and 8 × 8) increase from preferred to nonpreferred textures, yielding a significant positive correlation.

We recorded the activity of 168 visually responsive neurons in IT cortex in response to 16 different textures presented at 5 array sizes. The responses of an example IT neuron to the full set of stimuli are depicted in Fig. 2. It can be seen that this neuron maintained its selectivity for texture across all array sizes. This was also evident from the significant positive correlations (average *r* = 0.83) between texture responses to adjacent array sizes (Fig. 2). To assess this effect across the population, for every pair of array sizes, we calculated the correlation between neuronal responses to the 16 textures. The average correlation across the population was positive for all pairs (*P* < 0.0005 for all pairs of array sizes, *t*-test), and nearly all the statistically significant correlations were positive (Fig. 3*A*). Thus IT neurons show congruent tuning for texture across array sizes.

To investigate the impact of array size on neuronal responses, we first calculated the average responses across all textures at each array size as a function of time. The resulting plots show that responses not only increased with array size but also became more phasic (Fig. 3*B*). To assess whether the impact of array size on the neuronal response depends on texture preference, for each cell we ranked textures from preferred to nonpreferred based on the average response across array size (in a 50- to 200-ms window after image onset). We then calculated the normalized response (normalized to the maximum response across all stimuli) as a function of array size for these ranked textures across the neuronal population. The resulting plot (Fig. 3*C*, *top*) reveals that the response to preferred textures increases sharply with array size and saturates for larger arrays. In contrast, the response to nonpreferred textures increases monotonically with array size throughout.

To quantify these effects, for each texture (preferred to nonpreferred), we calculated the rate of increase per item relative to the maximum attained response across array size and plotted this rate of increase as a function of texture rank. For small array sizes (1–16 items), the rate of increase was large for preferred textures and small for nonpreferred textures (*r* = −0.70, *P* = 0.0025; Fig. 3*C*, *bottom*). For larger array sizes (16–64 items), the rate of increase was small for preferred textures and large for nonpreferred textures (*r* = 0.67, *P* = 0.0045; Fig. 3*C*, *bottom*).

**Fig. 4.**
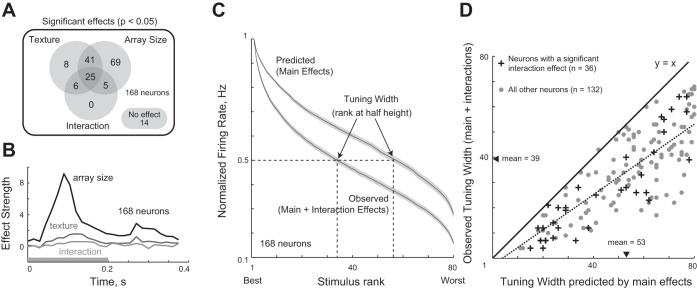
Effect of texture and array size on neuronal responses. *A*: distribution of the statistically significant effects in an ANOVA performed on the firing rate of each neuron with texture and array size as factors. The example neuron in Fig. 2 had a main effect of texture and array size but no interaction. *B*: time course of ANOVA effect strengths (averaged across all 168 neurons) for main effects of texture, of array size, and for interaction effects in 15-ms bins. *C*: normalized tuning curve (averaged across neurons) for the observed response and the response predicted from main effects alone. *D*: observed tuning width plotted against the tuning width predicted by main effects, for cells with significant interactions (crosses, *n* = 36) and all others (gray circles, *n* = 132). The dashed line is the best-fitting line and the solid line is the unit slope line.

To investigate the relative impact of texture and array size on the neuronal response, we performed an ANOVA on the firing rate of each neuron (50–200 ms after stimulus onset) with texture (16 levels) and array size (5 levels) as factors (with a significance level of *P* < 0.05). The resulting distribution of significant main and interaction effects is shown in Fig. 4*A*. A majority of the neurons (83%: 140 of 168) exhibited a main effect of array size. About 48% (80/168) of the neurons showed a main effect of texture. A relatively smaller fraction (21%: 36/168) of neurons showed a significant interaction effect (i.e., Fig. 4*A*).

To investigate the strength and time course of these effects, we calculated unbiased estimates of the strength of texture, array size, and interaction effects as a function of time (see methods). The resulting plot (Fig. 4*B*) shows that array size effects were stronger (peak strength = 9.1 Hz) and arose earlier (peak = 90 ms). In contrast, texture effects were weaker (peak strength: 1.6 Hz) and peaked slightly later (peak = 120 ms). Interaction effects were even weaker (peak strength: 0.6 Hz); their peak was 37% smaller than the texture modulation and developed even later (peak = 135 ms). To assess whether these differences in latency are statistically significant, we performed a bootstrap analysis: we selected 100 neurons with replacement from the entire population and recalculated the peak latency of each effect and repeated this procedure 100 times to obtain bootstrap-resampled estimates of latency for each effect. We found a significant difference between the peak latency for the texture and array size effects (*t*-test, *P* < 0.00005), as well as between texture and interaction effects (*t*-test, *P* < 0.05). We obtained qualitatively similar results on varying the number of neurons or bootstrap samples. We conclude that interaction effects were not only less frequent but were weaker and developed later relative to the main effects.

Although interaction effects are weak in magnitude, they may still exert a significant influence on neuronal tuning to texture. In particular there may be two types of interactions: those that sharpen or broaden the selectivity due to main effects, or those that alter the selectivity due to main effects. We reasoned that if interactions sharpen or broaden the selectivity due to main effects, the rank-order correlation between the response predicted by main effects and the response predicted by main and interaction effects should be unchanged. Alternatively, if interactions alter the selectivity due to main effects, there should be a reduction in the rank-order correlation for neurons that exhibit interaction effects. To distinguish between these possibilities, for each neuron, we calculated the response predicted by main effects alone and compared this with the observed response (which includes both main and interaction effects). We then compared the rank-order correlations between main effect predictions and the observed responses for neurons that exhibited only main effects (*n* = 118 from Fig. 4*A*) and neurons that exhibited interactions (*n* = 36 from Fig. 4*A*). We found no statistically significant difference (mean rank order correlations: 0.69 for main effects only, 0.72 for interactions; *P* = 0.30, unpaired *t*-test). Thus interactions do not fundamentally alter neuronal tuning to textures.

To assess whether interactions sharpen or broaden neuronal tuning across textures, for each cell we compared the sharpness of neuronal tuning between the observed response (due to main and interaction effects) and the response predicted by main effects. To measure the sharpness of tuning, we sorted the 80 stimuli (16 textures × 5 array sizes) in descending order of firing rate and calculated the stimulus rank at which the response attained half its maximum. Using this measure, a highly selective neuron would have a sharper tuning width. Across the population, the average observed response had sharper tuning compared with the response predicted by main effects alone (Fig. 4*C*). To visualize this pattern on a neuron-by-neuron basis, we plotted the observed tuning width against the tuning width expected from main effects (Fig. 4*D*). For nearly all neurons, the observed tuning width was smaller than that predicted from main effects (mean observed tuning width = 53; mean predicted tuning width = 39). We conclude that interaction effects sharpen but do not fundamentally alter neuronal tuning for textures.

### Experiment 2: Uniform Textures (Psychophysics)

Having characterized neuronal responses to discrete textures in IT, we proceeded to assess whether texture perception in humans can be explained using texture representations in IT cortex. To this end, we measured texture discrimination in humans using the same set of textures as in *experiment 1*. A total of 16 subjects performed a texture discrimination task in which they had to find a 4 × 4 texture array embedded within a larger 16 × 16 texture array (see methods for details and Fig. 1*A* for example displays). The time taken by subjects to find the target texture was taken as a measure of similarity between the target texture and its surrounding (distracter) texture. Subjects were extremely consistent in their performance, as evidenced by a high correlation in average reaction times between two randomly chosen halves of the subjects (average split-half correlation: *r* = 0.99, *P* < 0.00005).

**Fig. 5.**
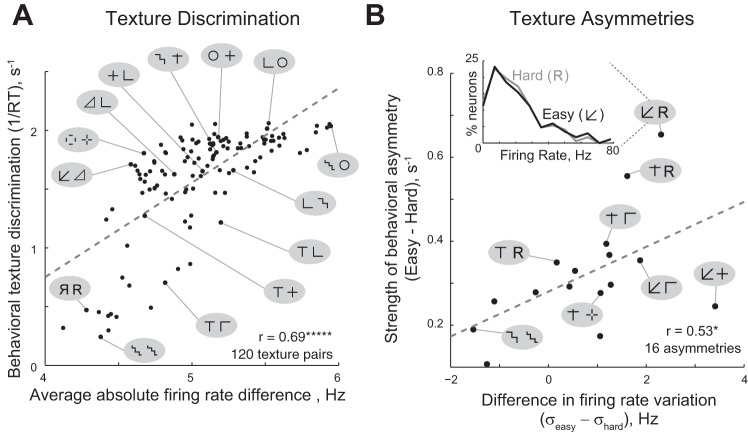
Texture discrimination in humans is predicted by monkey IT discriminability (*experiment 2*). *A*: texture discrimination in humans plotted against neuronal discriminability in monkey IT across all 120 pairs of textures tested. Example texture pairs are shown to illustrate the full range of the variation. The dashed line indicates the best-fit line. *B*: for pairs with a significant behavioral asymmetry in texture discrimination, we plotted the magnitude of the behavioral asymmetry against the difference in the standard deviation of firing rate across the population between the 2 textures of a pair. The dashed line indicates the best-fit line. *Inset*: Histogram of firing rates evoked by the easy (black) and hard (gray) targets in a texture pair with a large asymmetry (arrow/R). In all pairs (A, B), the first image of the pair is the easy target - in other words it is easier to find texture A embedded in B compared with finding B embedded in A.

To compare texture discrimination in humans with neuronal discriminability in IT cortex, we calculated a discriminability measure for each pair of textures (A, B) for both humans and IT neurons. For humans, because the average time to locate texture A within B (or vice versa, ignoring asymmetry for the time being) is a measure of similarity between the two textures, we took the reciprocal of this time to be a measure of texture discrimination in behavior. This 1/RT measure has a straightforward interpretation as a local saliency signal that accumulates to a threshold (Arun 2012; Vighneshvel and Arun 2013; Pramod and Arun 2014) and is correlated with neuronal discriminability in IT (Sripati and Olson 2010). For IT neurons, we took the average absolute difference in firing rate (|A−B|) (in a 50- to 200-ms window after image onset) across the population of neurons (across all array sizes) as a measure of neuronal discriminability. We observed a strong and significant correlation between neuronal and behavioral discriminability across all 120 textures tested (*r* = 0.69, *P* < 0.00005; Fig. 5*A*). To further confirm the robustness of this correlation, we calculated a d-prime measure of discriminability for each pair of textures both the neuronal and behavioral data (see methods). Here too we found a robust correlation between neuronal d-prime and behavioral d-prime (*r* = 0.45, *P* < 0.00005). We conclude that textures eliciting distinct patterns of activity across IT neurons are also highly discriminable for humans engaged in texture perception.

The above finding may be trivially explained by low-level similarity between images, because images similar at the pixel level may evoke similar activity even in the retina and therefore also in visual cortex. To assess this possibility, we asked whether texture discrimination in humans can be explained directly using image pixels instead of using monkey IT discriminability. For each pair of textures, we calculated the absolute difference in intensity between the two images averaged across pixels. We found a significant positive correlation between these pixel distances and behavioral discriminability (*r* = 0.53, *P* < 0.00005), but this correlation was smaller than the correlation observed between IT neurons and behavior (*r* = 0.69; Fig. 5*A*). This difference in correlation coefficients was statistically significant (*P* < 0.05, Fisher's *z*-test). Thus IT neurons explain texture discrimination better than image pixels (*r*^2^ = 48% for IT neurons and 28% for pixel distances).

Having established that IT texture representations explain human texture discrimination, we asked if they could explain a second related phenomenon, texture asymmetries. An example asymmetry is shown in Fig. 1*B*: it is easier to find the “arrow” texture among Rs than to find the R texture among the arrows. Can such asymmetries be explained using neuronal activity in IT? We began by first identifying asymmetries in texture discrimination in humans. For each texture pair (A, B), we performed an ANOVA on the response times with subject (16 levels), target texture (A/B as target) as factors. Texture pairs with a significant main effect of target texture were deemed as having an asymmetry (criterion *P* < 0.005). This procedure yielded 16 texture pairs with statistically significant behavioral asymmetries. For each such pair (A, B), if A was easier to find among B than B was in A, we denoted A as the easy target. We then obtained an unbiased estimate of the difference in discriminability between the easy and hard targets (see methods).

Next, we systematically tested plausible population measures of neuronal activity that predict the behavioral asymmetry. The simplest possibility is that texture A may be easier to find within texture B (than vice versa) if texture A elicited a stronger neuronal response on average compared with B. To test this idea, we calculated the difference in the average firing rate elicited by the easy and hard targets across neurons and asked if this difference could predict the behavioral asymmetry. This difference did not have a significant correlation with the behavioral asymmetry (*r* = 0.26, *P* = 0.33). Alternatively, we reasoned that texture A may be easier to find if it elicits greater variation in activity across neurons compared with B. To instantiate this idea, we calculated the difference in the standard deviation of the firing rates elicited by the easy and hard targets. This measure yielded a significant positive correlation with the behavioral asymmetries (*r* = 0.53, *P* = 0.037; Fig. 5*B*).

To further understand why the difference in standard deviation predicts the strength of asymmetry, we scrutinized the firing rate distributions for easy and hard textures for the asymmetric texture pairs. We reasoned that the standard deviation in firing rate will be large for a texture if a few neurons are driven strongly and others are driven only moderately. Thus the correlation between behavioral asymmetry and standard deviation differences may arise due to neurons that respond with high firing rates. To investigate this further, we limited our analysis to neurons with firing rates >50 Hz for each texture (typically ∼10 neurons/texture) and asked whether the behavioral asymmetry was correlated with the difference in average firing rates of the high firing neurons. This yielded a significant positive correlation (*r* = 0.67, *P* = 0.0042). Thus textures that elicit strong activity in a few neurons (but moderate activity in others) tend to produce asymmetries in behavior.

We conclude that both discrimination and asymmetries in human texture perception can be explained using neuronal activity in monkey IT.

### Experiments 3–4: Randomly Oriented Textures

Next, we investigated the third classic finding from human texture perception: for some texture pairs, the ease of discrimination reduces drastically when their elements are randomly oriented, but in other cases, it remains unchanged (Fig. 1*C*). Can texture representations in IT explain this finding? This also raises the general question of how responses to randomly oriented textures are related to that of uniform textures containing the individual orientations.

**Fig. 6.**
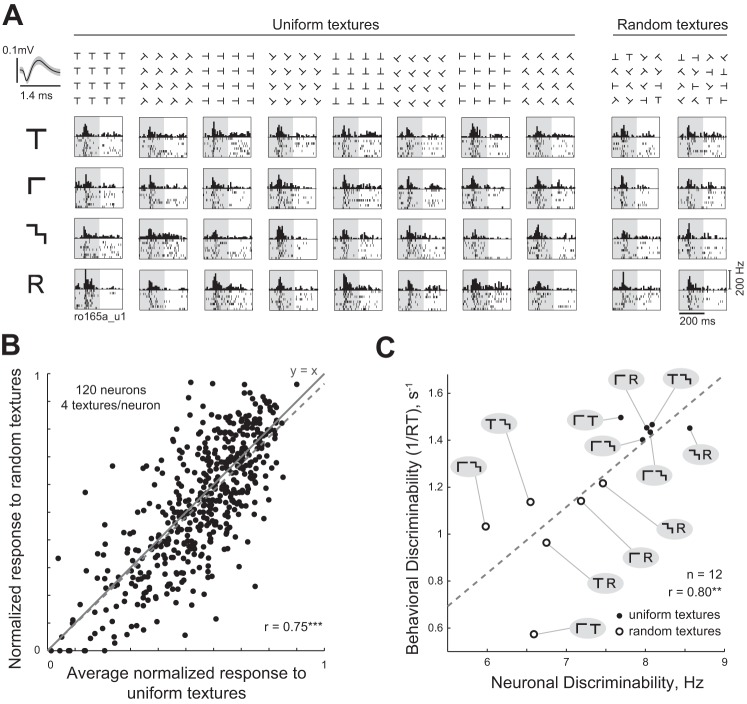
Relationship between uniform and random textures (*experiment 3*–*4*). *A*: responses of a sample neuron to uniform and randomly rotated textures. The 4 textures used are depicted in the *leftmost column*. Texture patches with elements at various orientations are shown for a sample shape (“T”) in the *topmost row*. Textures in the experiment were 8 × 8 arrays and are not shown in full due to space constraints. Each box represents the neuronal responses to the corresponding stimulus with conventions as in Fig. 2. *B*: for each neuron, the observed normalized response to the random textures is plotted against the average of responses across all orientations. This yielded a significant positive correlation (*r* = 0.75, *P* < 0.00005, dashed line). The thick gray line represents the *y* = *x* line. *C*: texture discrimination in humans plotted against neuronal discriminability in monkey IT for uniform and random textures. The dashed line indicates the best-fit line.

To this end, in *experiment 3*, we recorded the activity of 120 visually responsive neurons in response to uniform textures with elements in a single orientation and to textures with elements oriented randomly at each location (hereafter, random textures). Figure 6*A* illustrates the responses of an example IT neuron to the full stimulus set. This neuron responded distinctively to the uniform textures (average absolute difference in firing rate during 50–200 ms across all ^8^C_2_ pairs of orientations of each texture: 10.6 Hz) but similarly to the random textures (average absolute difference between the 2 random textures: 2.1 Hz).

How might random texture responses be related to the individual orientation responses? We reasoned that the response to many randomly oriented elements may be an average of the individual orientation responses as a result of multiple object normalization (Zoccolan et al. 2005). We therefore calculated a predicted response for each of the four random textures as the average of the eight uniform texture responses. We then plotted the observed responses to random textures against the predicted responses, the resulting correlation was positive and highly significant (*r* = 0.75, *P* < 0.00005; Fig. 6*B*).

The above correlation may arise because neurons maintain their selectivity to a particular texture element across orientations, rather than because of averaging. To assess this possibility, we repeated the above analysis except this time we took the response of each cell (from 50–150 ms after image onset) across textures for a single orientation as the predicted response to the random textures. Across the eight orientations, this too yielded a significant positive correlation (*r* = 0.55, *P* < 0.0005), but this correlation was smaller than the correlation obtained from the averaging (*r* = 0.75 in Fig. 6*B*). Thus 54% of the variance (0.55^2^/0.75^2^ = 0.54) in the response to random textures is explained using single neuron selectivity alone. Thus the remaining variance in the responses to random textures (i.e., at most 46%) can be attributed to an averaging of multiple orientation responses.

We then asked whether texture discrimination for the uniform and random textures in humans can be explained using neuronal discriminability in IT (*experiment 4*). To this end, we asked human subjects to perform a texture discrimination task as before using uniform and random textures (see methods). Subjects were consistent in their responses (split-half correlation in their reaction times was *r* = 0.61, *P* < 0.00005). Importantly, both IT neurons and humans in texture discrimination showed concordant patterns: across both uniform and random textures, texture discriminability in IT neurons was strongly correlated with discrimination behavior in humans (r = 0.80, *P* = 0.0017; Fig. 6*C*). We conclude that random rotations of texture elements renders textures less discriminable in IT neurons due to multiple object normalization and this leads to congruent reductions in texture discrimination in humans.

## DISCUSSION

The general conclusion of this study is that texture representations in monkey IT neurons predict texture discrimination in humans. In particular, we have found that *1*) IT neurons show congruent tuning for textures across array sizes, with interactions that serve to sharpen tuning; *2*) textures that elicit distinct neuronal activity in IT are also easily distinguished by humans engaged in texture discrimination; *3*) textures that elicit strong variations in activity across IT neurons tend to produce texture discrimination asymmetries in humans; and *4*) reduced discrimination for randomly oriented textures in behavior is predicted by concomitant reductions in IT neuronal discriminability, which in turn arises from averaging of uniform orientation responses. Below we discuss each of these findings in relation to the literature.

We first make explicit our assumption that an array of shapes is perceived as a texture. While this is a common assumption in studies of texture, it is impossible to test experimentally because there is no clear definition of what constitutes a texture as distinct from a collection of shapes (Adelson 2001). Likewise, there is no clear distinction between texture discrimination (i.e., finding a texture boundary) and visual search (i.e., finding a single shape) (Wolfe 1992). Our finding that IT neurons show congruent tuning for texture across array size (including for individual elements; Fig. 3*A*) underscores the possibility that responses to textures in IT neurons can be explained largely by selectivity to the shape of the constituent elements. However, texture selectivity influenced how responses changed with array size: responses to preferred textures increased sharply with array size and tended to saturate, whereas nonpreferred responses tended to increase monotonically. One possible underlying mechanism could be that strong excitatory drive (from preferred textures) recruits feedforward inhibition whereas weaker excitatory drive (from nonpreferred textures) does not. Alternatively, a strong excitatory drive may put the neuron in a high conductance state, leading to reduced gain, whereas a weak excitatory drive produces linear summation (Chance et al. 2002).

We have found that texture discriminability in monkey IT predicts human texture perception. The simplest interpretation of this neuronal-behavioral correlation is that texture discrimination in humans is based on texture representations similar to those we have observed in monkey IT (Sripati and Olson 2010). However, this is an indirect inference: the neuronal-behavioral correlation most definitely does not prove that IT neurons participate in texture perception in monkeys. Establishing this will require measuring neuronal activity in IT while monkeys perform a texture discrimination task. The neuronal-behavioral correlation we have found must be carefully interpreted while taking into account several important differences between the neuronal and behavioral data. The neuronal data were recorded while monkeys fixated the textures without discriminating them. The behavioral data were recorded while humans located a target texture embedded inside a larger texture with no constraints on their eye movements.

Given these differences, the fact that we have observed any correlation at all between monkey IT and human behavior is surprising, because it could have been abolished by at least four possible factors. *1*) Texture representations in humans and monkeys may be qualitatively different, but in fact, there is a growing body of evidence that neuronal representations in monkey IT are similar to those in human LOC (Kriegeskorte et al. 2008) and that they can explain various aspects of human vision (Sripati and Olson 2009; Sripati and Olson 2010). This is also corroborated by the fact that monkeys and humans show similar performance during object discrimination and categorization (Op de Beeck et al. 2001; Sigala et al. 2002). *2*) Neuronal representations during active task discrimination may be qualitatively different from those observed during fixation, but in fact, IT neurons show similar responses when monkeys switch between discrimination and fixation tasks (Suzuki et al. 2006). Even when neurons are modulated by attention, they undergo changes in gain but maintain their selectivity (Martinez-Trujillo and Treue 2004; McAdams and Maunsell 1999). *3*) Texture discrimination may be driven by interactions between the two textures at their common boundary (Nothdurft et al. 2000). *4*) Texture discrimination may be driven by integrating visual information across eye movements. Despite these factors, our finding of a robust, positive behavioral-neuronal correlation has two implications: *1*) it places limits on the extent to which the above factors influence texture discrimination, and *2*) it suggests that the correlation would probably be even larger if both the neuronal and behavioral data were collected simultaneously.

Our results explain three important observations in human texture perception using neuronal activity in monkey IT. First, our finding that textures eliciting distinct patterns of activity in IT are also easy for humans engaged in texture discrimination (Fig. 5*A*) is concordant with a similar observation regarding visual search (Sripati and Olson 2010). It suggests that visual search and texture discrimination might be based on similar underlying representations. It is possible that neuronal activity in areas presynaptic to IT may also explain texture discrimination; this is consistent with the fact that computational models of V1 neurons explain texture discrimination at least for a subset of the texture pairs tested here (Malik and Perona 1990).

Second, we have found that asymmetries in texture perception occur when one of the textures in a pair elicit a large variation in activity across IT neurons. Although asymmetries in texture perception have been studied extensively (Treisman and Souther 1985; Gurnsey and Browse 1987; Treisman and Gormican 1988; Gurnsey et al. 1992; Williams and Julesz 1992; Wolfe 2001), their neuronal correlates have never been investigated. Here we have for the first time demonstrated a plausible neuronal correlate for asymmetry. One interpretation of our finding is that textures that elicit strong activity in a few neurons become more discriminable. However, the generality of this finding remains to be established, because it is based on testing only a few asymmetries. However, texture asymmetries are themselves relatively rare: among the 120 texture pairs tested, only 13% had asymmetries. This is concordant with our recent finding that the occurrence of asymmetries in visual search is also relatively low (Arun 2012).

Finally, our results explain in neural terms the classic finding that texture discrimination becomes harder in some cases for random textures (Beck 1972; Julesz 1986; Malik and Perona 1990). Neuronal responses to random textures were accurately predicted by an average of their responses to the individual texture elements (Fig. 6*B*). This finding is consistent with the averaging response observed in IT when multiple objects are present in the receptive field (Zoccolan et al. 2005). As a result, the random textures become less discriminable in IT neurons, which predicted the reductions in discrimination observed during behavior (Fig. 6*C*). This is concordant with the fact that visual clutter (i.e., multiple objects) reduces discriminability for distinct items in IT (Miller et al. 1993; Missal et al. 1999; Zoccolan et al. 2005, 2007; Zhang et al. 2011).

Taken together, our results elucidate the neuronal representation of discrete textures in IT and suggest that texture perception in humans is based on texture representations similar to those observed in monkey IT.

## GRANTS

This research was funded by a start-up grant from the Indian Institute of Science, the DBT-IISc partnership programme, and an Intermediate Fellowship from the Wellcome Trust–DBT India Alliance.

## DISCLOSURES

No conflicts of interest, financial or otherwise, are declared by the author(s).

## AUTHOR CONTRIBUTIONS

Author contributions: K.A.Z. and S.P.A. conception and design of research; K.A.Z. and S.P.A. performed experiments; K.A.Z. and S.P.A. analyzed data; K.A.Z. and S.P.A. interpreted results of experiments; K.A.Z. and S.P.A. prepared figures; K.A.Z. and S.P.A. drafted manuscript; K.A.Z. and S.P.A. edited and revised manuscript; K.A.Z. and S.P.A. approved final version of manuscript.
